# Emotional Regulation in Teens and Improvement of Constructive Skills (EmoTIConS): study protocol for a randomized controlled trial

**DOI:** 10.1186/s13063-021-05886-2

**Published:** 2021-12-14

**Authors:** Laura Pedrini, Roberta Rossi, Laura Rosa Magni, Mariangela Lanfredi, Serena Meloni, Clarissa Ferrari, Ambra Macis, Nicola Lopizzo, Valentina Zonca, Annamaria Cattaneo

**Affiliations:** 1 Psychiatry Unit, IRCCS Istituto Centro San Giovanni di Dio Fatebenefratelli, Brescia, Italy; 2 Statistics Service, IRCCS Istituto Centro San Giovanni di Dio Fatebenefratelli, Brescia, Italy; 3 Biological Psychiatry Unit, IRCCS Istituto Centro San Giovanni di Dio Fatebenefratelli, Brescia, Italy; 4 Department of Pharmacological and Biomolecular Sciences, University of Milan, Milan, Italy; 5 Department of Psychological Medicine, Institute of Psychiatry, Psychology & Neuroscience, King’s College London, London, UK

**Keywords:** Emotional dysregulation, Adolescents, Socioemotional learning, School, Stress, Cortisol, Randomized controlled trial

## Abstract

**Background:**

Emotional dysregulation (ED) constitutes a relevant factor involved in the onset and maintenance of many mental disorders. Targeting ED during adolescence could be a determinant both to identify high-risk individuals and to promote preventive interventions. This study will aim to evaluate the impact of a brief Dialectical Behavioral Therapy (DBT)-based intervention for adolescent students by measuring changes in emotional regulation skills and impulsive behaviors. Moreover, alterations in biological features related to stress response and inflammation will be assessed as potential biological variables associated with ED.

**Methods:**

This is a randomized trial. A total of 20 classes of adolescent students will be recruited among high schools in Brescia, a city in northern Italy. They will be randomized to the psychoeducational intervention (experimental group) or to a control condition (control group). The intervention will be based on DBT Skills Training for Emotional Problem Solving for Adolescents, and will consist of four monthly, 2-h sessions (for a total of 8 h) scheduled during regular school time. Participants will be assessed at baseline, post-intervention, and at 3 and 6 months of follow-up. The primary outcome measures will be represented by changes in the use of emotional regulation skills and by changes in the frequency of impulsive behaviors. Salivary samples will be collected at baseline and post-intervention to explore possible biological features underlying ED.

**Discussion:**

Data from the present project will offer the opportunity to better understand the complex phenomenon of ED. Repeated assessment will cover several domains (emotional, behavioral, social, biological) as potential factors associated with ED. Moreover, it will be possible to establish the effect of the proposed intervention, thus helping to improve knowledge on the impact of school-based universal preventive programs. Finally, the current trial will propose an integrated screening and intervention-based model. Ultimately, this could reduce barriers to youths’ mental health care by fostering collaboration between schools and mental health services.

**Trial registration:**

ClinicalTrials.gov NCT04349709. Registered on April 16, 2020.

## Administrative information

Note: the numbers in curly brackets in this protocol refer to SPIRIT checklist item numbers. The order of the items has been modified to group similar items (see http://www.equator-network.org/reporting-guidelines/spirit-2013-statement-defining-standard-protocol-items-for-clinical-trials/).

**Table:** 

Title {1}	Emotional Regulation in Teens and Improvement of Constructive Skills (EmoTIConS): Study protocol for a randomized controlled trial.
Trial registration {2a and 2b}.	ClinicalTrials.gov Identifier: NCT04349709
Protocol version {3}	ClinicalTrials.gov Registered 16/04/2020
Funding {4}	This trial was funded by the Italian Ministry of Health in the framework of the grant “BANDO 2018 GIOVANI RICERCATORI e RICERCA FINALIZZATA” (GR-2018-12366754).
Author details {5a}	Laura Pedrini ^a^, Roberta Rossi ^a^, Laura Rosa Magni ^a^, Mariangela Lanfredi ^a^, Serena Meloni ^a^, Clarissa Ferrari ^b^, Ambra Macis ^b^, Nicola Lopizzo ^d, e^, Valentina Zonca ^d, f^, Annamaria Cattaneo ^d, e^ ^a^ Psychiatry Unit, IRCCS Istituto Centro San Giovanni di Dio Fatebenefratelli, Brescia, Italy. ^b^ Statistics Service, IRCCS Istituto Centro San Giovanni di Dio Fatebenefratelli, Brescia, Italy. ^d^ Biological Psychiatry Unit, IRCCS Istituto Centro San Giovanni di Dio Fatebenefratelli, Brescia, Italy. ^e^ Department of Pharmacological and Biomolecular Sciences, University of Milan, Italy. ^f^ King's College London, Department of Psychological Medicine, Institute of Psychiatry, Psychology & Neuroscience, London, UK
Name and contact information for the trial sponsor {5b}	IRCCS Istituto Centro San Giovanni di Dio, via Pilastroni 4, 25125 Brescia
Role of sponsor {5c}	It is responsible for the arrangements and implementation of the trial.

## Introduction

### Background and rationale {6a}

Adolescence is a critical period since most mental disorders develop before the age of 25 [1]. The Health Behavior in School-aged Children (HBSC) study prospectively assessed several aspects of the physical and psychological well-being of adolescent students from different countries [2]. Findings from the HBSC showed a small, linear increasing trend in psychological distress indices, especially in higher-income countries [3]. Of note, compared to other countries, Italy reports the highest rate of youths that refer to sadness, irritability, sleep problems, and psychosomatic health complaints [3].

The ability to regulate emotions represents a determinant variable and is strictly associated with the mechanisms involved in pursuing personal goals and establishing positive interpersonal relationships [4]. Difficulties in emotional regulation processes represent a relevant risk factor for the development of mental disorders [5, 6]. Emotional dysregulation (ED) is a multidimensional construct including different facets, such as a lack of awareness about experienced emotions, non-acceptance of emotional distress, impulsivity, the inability to pursue goals when emotionally distressed, and a lack of regulatory strategies [4]. ED embodies the core dimension of borderline personality disorder (BPD) and is mainly characterized by rapid and intense emotional changes, impulsive behaviors, and unstable relationships [7]. The reduced ability to regulate emotions denotes a relevant feature that can be observed in other mental disorders, such as depression and anxiety [8, 9], and with other clinical conditions, such as substance-related issues [10, 11], suicidal ideation [12], and self-harm [13]. Indeed, in the absence of more adaptive coping mechanisms, health-risk behaviors (e.g., self-harm, alcohol or substance abuse, risky sexual behaviors) are sometimes used to manage psychological distress [14, 15].

Since emotional regulation is a modifiable skill, interventions helping students to improve emotional regulation-related strategies could represent a promising primary prevention approach. Several studies support the use of socioemotional learning (SEL) as part of school educational programs [16, 17]. SEL programs aim to help children and adolescents acquire and practice the skills they need to successfully cope with stressful life events [16, 17]. A meta-analysis of SEL programs found that children and adolescents who participated in SEL programs showed better indicators of social-emotional skills and well-being than those who did not participate [18]. Adolescence is characterized by high emotional stress because of demands that typically occur during this period such as academic pressure, dating and intimacy issues, bullying, peer rejection, alcohol and drug use, concerns about physical attractiveness, and becoming more independent of one’s parents. Therefore, adolescents may have a particular need to regulate their emotions to face stressors and to carry out crucial steps involved in forming their identity and personality [19].

There are different kinds and formats of school-based interventions to strengthen emotional regulation in adolescents. Available studies reveal a positive effect on mental health and emotional regulation skills; in contrast, there is scant evidence about the impact on maladaptive behaviors. Moreover, most interventions are not manualized, and most existing studies are not based on sample size calculations, nor do they provide follow-up assessments. It is still not possible to draw any definitive conclusions regarding the superiority of one intervention over another. However, DBT has several values that support its application. First, it is in line with the SEL criteria [16]; moreover, it is manualized and includes a broad range of cognitive and behavioral techniques. Finally, this program can be conducted by schoolteachers who have received the proper training.

Dialectical behavioral therapy (DBT) is an empirically supported treatment rooted in the biosocial theory of ED [20]. The common underlying dysfunction in emotional regulation makes this treatment a valid transdiagnostic approach in clinical settings [21]. DBT was originally conceived for adults with BPD; subsequently, this treatment was adapted for adults with different psychiatric conditions [22–24], as well as for adolescent patients [21, 25, 26]. DBT skills encompass basic social and emotional life competencies; indeed, they are helpful for everyone and are useful in non-clinical populations such as school contexts [27]. DBT STEPS-A is the adaptation of DBT specifically designed for students in middle and high school. Studies evaluating the efficacy of DBT STEPS-A are ongoing; however, preliminary data support its use in schools. Indeed, positive results have been found with a selected population of students experiencing behavioral or academic challenges [28]. Recently, DBT has been applied as a universal program for the entire class, and students have exhibited improvement in social resiliency and emotional dysregulation [29].

Emotional regulation abilities develop substantially during adolescence, shifting from reliance on parental support to internal regulatory processes [30]; this transformation parallels the neurobiological changes in brain circuits involved in emotional processes [31]. It is well known that early traumatic experiences and stressful life events are strong risk factors for the onset of mental disorders [32], and individuals exposed to early traumatic experiences display difficulties in regulating their emotional responses [33]. Among the biological systems involved in the effect of stressful experiences on the vulnerability to develop ED and psychiatric disorders more generally, there is the hypothalamic-pituitary-adrenal (HPA) axis [34–36]. More specifically, exposure to early stressful life events may cause persistent alterations in the HPA axis’ functionality, leading to a persistent release of the stress hormone cortisol. Cortisol acts on its receptors, which are widely distributed in limbic brain regions, thus influencing stress response, cognition, affect, and behavior [34].

In line with this, in a sample of 180 adolescents at high risk for psychopathology, it was found that high morning cortisol levels predicted the onset of a major depressive episode in the following year [37]. Another, similar study indicated that high morning saliva cortisol levels predicted the development of depressive symptoms by the age of 16 in 13-year-old adolescents [38]. However, although the presence of alterations in cortisol release and depression is well established, weaker outcomes are found in the context of anxiety disorder or externalizing disorder. Indeed, some authors did not find an association between cortisol and psychopathology, or discovered an association in a different direction than expected [39, 40]. The age and severity of symptoms constitute relevant factors for HPA axis functioning, thus contributing to explaining the inconsistencies in the available evidence [39]. Moreover, the majority of the studies included adults.

Investigations with adolescents are needed to understand the exact nature of the relationship between the HPA axis and psychopathology. In this framework, the identification of biological variables linked to difficulties in emotional regulation in youths has been recognized as a relevant domain to identify possible factors of susceptibility to mental disorders [41].

### Objectives {7}

The aim of the present study is twofold. The first is to evaluate the impact of a school-based intervention focused on emotional regulation strategies in a sample of adolescent students. The primary outcome will be changes in the frequency of use of emotional regulation skills and changes in the frequency of impulsive behaviors. Our hypothesis is that (1) at the end of intervention, the students who received the intervention will report a reduction in the frequency of impulsive behaviors compared to baseline, whereas we expect no differences in the group of adolescents who will not receive the intervention; (2) at the end of intervention, the students will report an increase in the use of emotional regulation skills compared to baseline, while we expect no differences in the control group; and (3) these changes will be maintained at 3 and 6 months of follow-up.

The second aim was to assess the association between biological features and ED in adolescent students. Specifically, we will compare several biomarkers related to stress response and inflammation in subgroups of students with different levels of ED assessed with the Difficulties in Emotion Regulation Scale (DERS) [4] measured at baseline. Moreover, the repeated collection of salivary samples post-intervention will allow us to evaluate the role of these biological features in the interventions’ effects on the behavioral variables described above (i.e., impulsive behaviors and use of skills).

### Trial design {8}

This is an interventional, parallel assignment, two-arm, cluster randomized trial. The classes of students will be randomized into a psychoeducational intervention (experimental group) or control condition (control group). All participants will be assessed at baseline, post-intervention, and at the 3- and 6-month follow-up points by using self-report questionnaires. In addition, salivary samples will be collected at baseline and post-intervention. Details on the timeline and assessments are reported in Table 1.

**Table 1:**
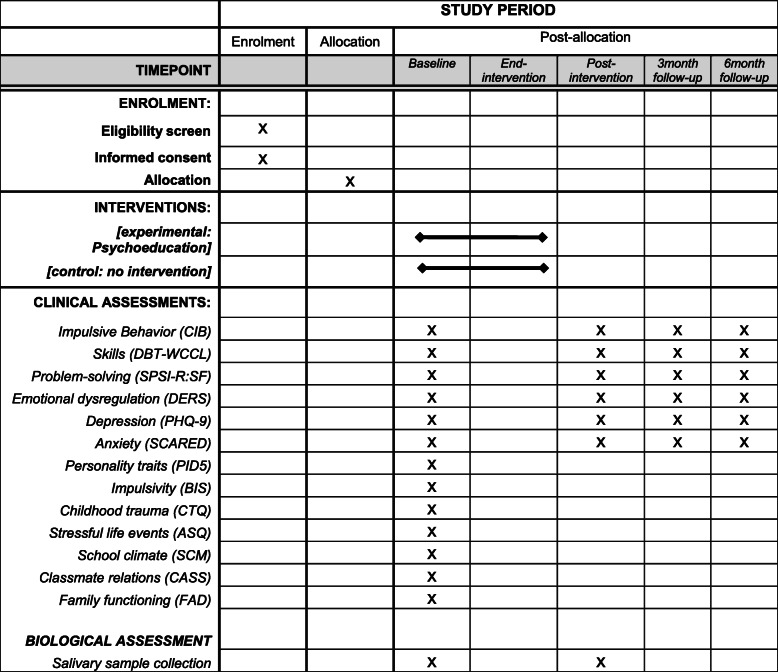
Timeline and assessments of the study

*CIB* Checklist of Impulsive Behaviors, *DBT-WCCL* DBT-Ways of Coping Checklist (DBT-WCCL), *SPSI-R:SF* Social Problem-Solving Inventory-Revised Short Form, *DERS* Difficulties in Emotion Regulation Scale, *PHQ-9* Patient Health Questionnaire, *SCARED* Screen for Child Anxiety Related Emotional Disorders, *PID5* Personality Inventory for DSM-5, *BIS-Brief* Barratt Impulsiveness Scale–Brief, *CTQ* Childhood Trauma Questionnaire, *ASQ* Adolescent Stress Questionnaire, *SCM* School Climate Measure, *CASS* Child and Adolescent Social Support Scale, *FAD* Family Assessment Device

The present study protocol was written in accordance with the Standard Protocol Items: Recommendations for Interventional Trials (SPIRIT) [42].

## Methods: participants, interventions, and outcomes

### Study setting {9}

A total of 20 school classes will be recruited among the high schools of Brescia (Northern Italy), for a total of approximately 440–460 students (considering approximately 22–23 students per school class) attending several high schools in the city. Each school will enroll an even number of classes to be randomized to the experimental or control conditions.

### Eligibility criteria {10}

The inclusion criteria will be as follows: 16–19 years; attending the third year of high school (10th grade); and signing an informed consent form. Exclusion criteria will be a certified diagnosis of learning difficulties or of autism spectrum disorders. All students completed the assessment questionnaires and participated in the project activities.

### Who will take informed consent? {26a}

Participation in the study will be voluntary, and the participants will be allowed to withdraw at any time. Teachers and project staff members will organize meetings with parents and students to introduce the study and to answer their questions. Project staff members will ask both students and parents to sign the informed consent form. Information sheets and consent forms (both for parents and students) were approved by the local ethics committee, the Comitato Etico IRCCS Fatebenefratelli.

### Additional consent provisions for collection and use of participant data and biological specimens {26b}

This trial involves collecting biological specimens of saliva to measure stress and inflammation. On the consent form, participants will be asked to state if they agree to this part of the trial or if they prefer to withdraw from it. As for the other parts of the trial, the subjects will be informed that they can withdraw at any time. In this case, biological samples will be destroyed.

## Interventions

### Explanation for the choice of comparators {6b}

All activities of the trial, both the assessments and the intervention, will be done during school time instead of the usual schooling curricula. Hence, the classes randomized to the control condition will follow their usual schooling curricula and will receive the intervention after the follow-up. All this information is reported in the consent forms. The reasons for our choice are based on the consideration that we did not find an intervention to be as effective as a standard. Moreover, the results of our trial will be useful to support the inclusion of specific curricula in school scheduling, as they are still lacking in our country.

### Intervention description {11a}

The psychoeducational intervention will be based on DBT Skills Training for Emotional Problem Solving for Adolescents (DBT STEPS-A), a manualized program designed to help adolescent students develop coping strategies and decision-making abilities, especially under emotional distress [43]. The DBT STEPS-A covers all four primary skills modules of the DBT skills training (mindfulness, distress tolerance, emotional regulation, and interpersonal effectiveness). The DBT STEPS-A program was created in the US context to be delivered as a universal social–emotional learning curriculum; it consists of approximately 30 weekly lessons organized into 50-min blocks.

For the present trial, we selected within the original DBT STEPS-A the specific elements related to ED. The intervention will consist of four sessions of 2 h each per month (for a total of 8 h) scheduled during regular school time. The details of each session are reported in Table 2. Sessions will be conducted by two psychotherapists (a leader and a co-leader) who have attended specific trainings on DBT [20] and DBT-A [44].

**Table 2:** Description of the intervention

	Aims	Contents and skills	Methods^a^
Session 1 (2 h)	1) To describe goals of emotional regulation and function of emotions 2) To enhance emotional literacy	• Emotional dysregulation and emotional regulation skills. • Levels of emotion chart • The concept of biological vulnerability • The functions of emotions • Homework: to describe emotional events through the ABC (Antecedents, Beliefs, Consequences) diary.	- Slideshow - Handouts 15.1, 15.3 - Class exercices
Session 2 (2 h)	1) To practice with the model of emotions 2) To gain the skills to manage interpretations and impulses to action.	• The model of emotions: emotion is a complex response made up of different components (prompting event, interpretation, biological changes, expressions, and aftereffects). • Emotion are set off by our interpretation, not by the events themselves: the skill “Check the facts” • Emotion have an action urge, by changing behavior, we can change emotion: the skill “Opposite action” • Homework: practice with check the facts and opposite action	- Slideshow - Role-playing - Handouts 16.1-2, 17.2-4 - Class exercises
Session 3 (2 h)	1) To improve strategies to solve a problem 2) To learn how to accumulate positive emotions.	• How to solve a problem: the seven steps of problem-solving • Accumulate positive experiences in the short term: the skills “pleasant activities” and “building mastery”. • Accumulate positive experiences in the long term: identifying personal values and plan goals. • Vulnerability factors and the PLEASE skills • Homework: practice with AB Please	- Slideshow - Handouts 18.1, 19.2-5, 20.1-4 - exercises in small groups
Session 4 (2 h)	1) To learn how to reduce intense emotion quickly in order to avoid impulsivity	• The autonomic nervous system and the “fight-or-flight” reaction • Activate parasympathetic system by reducing body temperature, using intense exercise, and engaging in paced breathing: the TIPP skills. • Homework: practice with TIPP skills	- Slideshow - Handouts 6.2, 8.1. - Classes exercices.

^a^The handouts were taken from the Italian version of DBT STEPS-A 44

Active participation of students will be encouraged, and at the end of each session, the key concepts will be written on a poster hanging in the classroom as a reminder. Homework will also be assigned for students to practice the learned skills in a real environment and will be reviewed at the beginning of the following session.

### Criteria for discontinuing or modifying allocated interventions {11b}

There will be no special criteria for discontinuing or modifying the allocated intervention.

### Strategies to improve adherence to interventions {11c}

All activities of the study will be scheduled during regular school time according to academic planning to facilitate students’ attendance.

The contents of the psychoeducational intervention will be structured. The fidelity to the intervention will be supported by regular meetings of the project team. Team meetings will be scheduled weekly during the pre-study phase and will then be scheduled monthly. For each session of the psychoeducational intervention, the co-leader will fulfill a checklist to register the content of the session and to record students’ attendance.

Adherence to the procedure for salivary sample collection will be promoted by phone alert messages at established hours and by written reminders about the exact procedure.

### Relevant concomitant care permitted or prohibited during the trial {11d}

Concomitant care will be permitted.

### Provisions for post-trial care {30}

There is no anticipated harm or compensation for trial participation.

### Outcomes {12}

#### Primary outcomes

##### Emotional regulation skills

DBT-Ways of Coping Checklist (DBT-WCCL) [45]

The DBT-WCCL is a 59-item self-report questionnaire measuring the frequency of DBT skills use (DBT Skills Subscale, 38 items) and dysfunctional coping strategies (Dysfunctional Coping Subscale, 21 items). Participants will be asked to indicate, on a 4-point Likert scale, the frequency of use of each skill in the previous month (i.e., never used, rarely used, sometimes used, or regularly used). The scale showed good internal consistency (Cronbach’s alpha coefficients for the subscales ranged from .84 to .96) and content validity [45]. For our primary outcome analysis, we will assess the difference between the two groups in terms of the proportion of participants categorized as “improved” post-intervention. Specifically, we will consider as “improved” those participants with a difference of at least one category, toward improvement, from the baseline to post-intervention in the frequency of use of functional skills. Intervention efficacy will be defined by a difference of at least 20% (i.e., 12/59 items) of improvement between groups after the intervention.

##### Impulsive behaviors

Checklist for Impulsive Behaviors (CIB)

This CIB is an ad hoc 15-item checklist created to determine the frequency of impulsive behaviors: alcohol use (4 items), substance use (2 items), internet addiction (4 items), gambling (1 item), non-suicidal self-injury (2 items), unprotected sex (1 item), and binge eating (1 item). Participants will be asked to indicate, on a 5-point Likert scale, the frequency of impulsive behaviors that occurred in the previous month (never; only once a week; two or three times a week; at least 4 times a week). Moreover, the checklist includes a section to evaluate the lifetime frequency of each behavior on a 6-point Likert sale (1–2 times, 3–5 times, 6–9 times, 10–19 times, 20–39 times, at least 40 times). For our primary outcome analysis, we will assess the difference between the two groups in the proportion of participants categorized as “improved” at post-intervention. Specifically, we will consider as “improved” those participants with a difference of at least one category toward improvement from baseline to post-intervention in the frequency of impulsive behaviors. Intervention efficacy will be established by treatment success, defined as at least 13% (i.e., 2/15 item) between groups after the intervention.

#### Secondary outcomes

Secondary outcomes will be assessed in terms of differences in the mean score between baseline and post-intervention on the instruments measuring the following domains:

##### Problem-solving style

Social Problem-Solving Inventory-Revised Short Form (SPSI-R:SF) [46, 47]

The SPSI-R:SF is a 25-item, self-report questionnaire measuring abilities in problem-solving: positive problem orientation (PPO), negative problem orientation (NPO), rational problem-solving (RPS), impulsivity-carelessness style (ICS), and avoidant style (AS). Each item is rated on a 5-point Likert scale, and scores are computed for each of these subscales, as well as a global score for total inventory. Higher scores on the NPO, ICS, and AS reflect a more maladaptive approach to problem-solving, whereas higher scores on the PPO and RPS indicate more adaptive strategies. Studies have reported good construct validity, internal consistency (with Cronbach’s alpha coefficients ranging from .73 to .86), and test–retest reliability (with the intraclass correlation coefficients ranging from .74 to .87) [46, 47].

##### Emotional dysregulation

Difficulties in Emotion Regulation Scale (DERS) [4, 48]

The DERS is a 36-item self-report questionnaire measuring different dimensions of ED: non-acceptance of emotional responses; difficulties engaging in goal-directed behavior; impulse control difficulties; a lack of emotional awareness; limited access to emotional regulation strategies; and a lack of emotional clarity. Each item is rated on a 5-point Likert scale, and scores are computed for each of these subscales, as well as a global score for total inventory. The DERS is the most comprehensive measure of ED to date and exhibits good reliability and validity when administered to adults [4]. Specific analyses also confirmed the reliability and validity of the DERS in adolescents (Cronbach’s alpha coefficients ranged from .76 to .89 for the subscales) [49]. The Italian version of the DERS showed adequate and comparable properties [48].

##### Depression

Patient Health Questionnaire-9 (PHQ-9) [50]

The PHQ-9 is a 9-item, self-report questionnaire measuring depression severity. Each of the 9 items can be scored from 0 (not at all) to 3 (nearly every day); a total score is computed as the sum of the nine items. Studies have shown that the PHQ-9 has excellent internal validity (with Cronbach’s alpha coefficient higher than .85) and test–retest reliability. Different levels of severity of depression can be distinguished according to the cutoff [50].

##### Anxiety

Screen for Child Anxiety Related Emotional Disorders (SCARED) [51, 52]

The SCARED is a 38-item, self-report questionnaire measuring different manifestations of anxiety: panic disorder, generalized anxiety disorder, separation anxiety disorder, school anxiety, and social anxiety. The SCARED showed good internal consistency and test–retest reliability [51]. The Italian version of the SCARED was found to be a valid screening instrument [52].

##### Impulsivity

Barratt Impulsiveness Scale–Brief (BIS-Brief) [53]

The BIS-Brief is an 8-item, self-report questionnaire that establishes the personality construct of impulsiveness in adolescents. The BIS-Brief showed good reliability and construct validity [53].

At baseline, data on sociodemographic data—such as information on number of visits with mental health professionals, pharmacological treatments, lifestyle (sleep quality, leisure activities, number of friends), and academic performance—will be collected. In addition, at baseline, the following instruments will be administered to assess psychosocial domains associated with ED: the Personality Inventory for DSM-5 [54] for personality traits; the Childhood Trauma Questionnaire [55] for early adverse experiences; the Adolescent Stress Questionnaire [56] for stressful life events in the previous year; the School Climate Measure [57] for school climate; the Child and Adolescent Social Support Scale [58] for relationships with classmates; and the Family Assessment Device [59] for family functioning.

### Participants’ timeline {13}

Details about the timeline and assessments are reported in Table 1.

### Sample size {14}

The primary outcome measures will be represented by changes in impulsive behaviors (established by the Checklist for Impulsive Behaviors) and by changes in emotional regulation skills (determined by the DBT-Ways of Coping Checklist).

Precisely, the outcome will be evaluated in terms of change (pre/post) in percentage of “yes” answers in the two groups (treated and control), where “yes” is defined as a difference of at least one category toward improvement from baseline. More specifically, the scores of the Checklist for Impulsive Behaviors are measured on a Likert scale. Therefore, a “yes” answer implies a decrease in the frequency of impulsive behavior; for example, from the category “two or three times a week” to the category “one time a week.” In a similar manner, a “yes” answer for the DBT-Ways of Coping Checklist means a decrease in the use of dysfunctional skills; for example, from the category “regularly used” to “sometimes used,” or vice versa for functional skills.

We assumed that at baseline, there would be a between-group difference by chance of 7% (i.e., 1/15 yes answers, where 15 is the total number of checklist items) on the Checklist for Impulsive Behaviors and that this difference would increase by up to 13% (i.e., 2/15 yes answers) after the intervention. Based on these assumptions and applying a two-sided test for paired binomial proportions, with a significance level equal to 0.05 and a power of 0.8, the estimated sample size was *n* = 340 (170 per group). Moreover, considering a drop-out rate of 20%, the minimum sample size (MSS) reached *n* = 426 (213 per group).

In the same way, we assumed that on the DBT-Ways of Coping Checklist, there would be a difference by chance of 7% (i.e., 4/59 yes answers, where 59 is the total number of the checklist DBT items) at baseline, and that this difference would increase by up to 20% (i.e., 12/59 yes answers) after the intervention. This last hypothesis was made considering that, given the focus of treatment, we expected a greater change in emotional regulation skills than in dysfunctional behaviors. Based on these considerations and applying a two-sided test for paired binomial proportions, with a significance level equal to 0.05 and a power of 0.8, we reached an estimated sample size of *n* = 84 (42 per group).

In sum, due to both the outcome measures and related sample size estimations, the final sample size needed was 426 students.

### Recruitment {15}

The enrollment of the target sample size will be ensured by specific strategies based on loyalty. The research team has drawn upon already consolidated collaboration with several schools in the city. Moreover, all students completing all assessments will be awarded books or e-books.

## Assignment of interventions: allocation

### Sequence generation {16a}

After enrollment, the statistician will randomly assign the classes within each school to experimental or control conditions. More specifically, a random sequence of strings (of length equal to the number of school classes) with labels “Experimental” or “Control” will be generated by a computer code, with a ratio of 1:1 (experimental vs. control). The classes will then be linked to the random sequence following their alphabetic order.

### Concealment mechanism {16b}

Allocation concealment will be ensured since the statistician service will not release the randomization code until the classes of students have been recruited into the trial, which takes place after all baseline measurements have been completed.

### Implementation {16c}

The statistics service of IRCCS Fatebenefratelli San Giovanni di Dio will generate the allocation sequence, which will be stored in a digital database protected by passwords on a secure server at the study site. Only the statistician will have access to the list. The PI’s collaborators will be responsible for recruitment and will request the randomization code after the baseline assessment session. Then, the PI will inform the schoolteacher about the final assignment of each class.

## Assignment of interventions: blinding

### Who will be blinded {17a}

Given the nature of the intervention, neither the project psychologists nor the participants could be blinded to the intervention. Outcome assessment will be based on self-report questionnaires, and data analysts will be blinded.

### Procedure for unblinding if needed {17b}

The design is open label with only data analysts being blinded, so unblinding will not occur.

## Data collection and management

### Plans for assessment and the collection of outcomes {18a}

To enhance data validity, assessment sessions will be planned at school for each class of students at baseline, post-intervention, and at 3 and 6 months of follow-up. The researcher will remain available during the sessions to answer students’ questions and to ensure that students complete the questionnaires independently.

### Plans to promote participants’ retention and complete follow-up {18b}

To promote both involvement in the evaluation and to ensure that each participant can complete the entire evaluation, all activities will be planned in agreement with school personnel during the school period. Moreover, one or two teachers will be identified as the contact persons for each class. We did not set criteria to define drop-out; however, we registered attendance at the sessions for each participant. All participants will be invited to complete and follow-up questionnaires independent of attendance.

### Data management and storage {19}

Participants will complete the paper-based battery of questionnaires at school. At the end of the assessment session, the researcher will collect the completed questionnaire and will bring the forms to the IRCCS Istituto Centro San Giovanni di Dio Fatebenefratelli. Data will be registered in a digital database that will be stored at the study site following procedures in line with privacy policies. In particular, after obtaining informed consent, each participant will be associated with an alphanumeric unique code, databases will be stored on a secure server, and they will be protected by passwords. All the material will be stored in a locked archive in areas with limited access. Only authorized research personnel will have access to the database. Quality data checks will be performed after each assessment session by the PI and research personnel to ensure that guidelines for data collection are followed.

### Confidentiality {27}

After obtaining informed consent, each participant will be associated with a unique alphanumeric code. The identification list of subjects will be kept in the investigator’s file and separated from other files. All electronic files will be stored on a secure server and protected by passwords. Only authorized research personnel will have access to the database. The researcher will provide the paper-based battery of questionnaires to each participant corresponding with his/her unique alphanumeric code. The data will be shared only upon reasonable request, to be evaluated by the PI.

### Workflow for collection, laboratory evaluation, and the storage of biological specimens for molecular analysis {33}

After completing the questionnaires, the researcher will explain the procedure to collect salivary samples via OraGene tubes and Salivette, which will be used to measure a panel of inflammatory mediators with Luminex technology. RNA samples from OraGene tubes will be isolated using RNAesy Kits, RNA samples will be processed to obtain a gene expression profile by employing the RNAseq technique on the Illumina platform, and the data will be analyzed to identify peripheral genes associated with ED. Tubes for diurnal cortisol sample collection that will be carried out the next day (morning, afternoon, evening) will also be provided to each individual, and saliva samples will then be analyzed for cortisol levels via ELISA. Information reporting all the detailed steps regarding the exact procedure to gather saliva samples for cortisol measurements will be provided. On this form, there is also a dedicated space that can be used by the students to annotate the exact time of sample collection and any problems that occurred during saliva collection. Salivary samples gathered with both Oragene and Salivette will be stored at − 20 °C until subsequent analysis. The salivary samples will be used only for the purpose reported in this protocol.

## Statistical methods

### Statistical methods for primary and secondary outcomes {20a}

Descriptive statistics (frequencies and percentages for categorical variables, and means and standard deviations for continuous variables) will be evaluated. To compare categorical variables, the chi-squared test will be used. To compare continuous variables between the different groups of interest, *t*-tests, ANOVA, or corresponding non-parametric tests (Mann–Whitney or Kruskal–Wallis) will be performed. Moreover, generalized mixed models will be harnessed to assess the differences in dysfunctional behaviors and emotional regulation skills across time and between groups (including post hoc analyses).

Differences in the levels of the biomarkers will be assessed by using linear and generalized linear models adjusting for potential confounders such as sex. Correlations (Pearson or Spearman) and generalized linear models will be employed to assess the association between the level of emotional dysregulation and other clinical variables and biomarkers.

### Interim analyses {21b}

There are no anticipated problems that are detrimental to the participants.

### Methods for additional analyses (e.g., subgroup analyses) {20b}

Analyses for primary and secondary outcomes will include (i) subgroup evaluation through post hoc analysis and (ii) adjustment for potential confounders, so no further additional analyses will be performed.

### Methods in analysis to handle protocol non-adherence and any statistical methods to handle missing data {20c}

An evaluation of the type of missing data will be performed to detect any missing not-at-random outcome data. In the case of missing not-at random or missing outcomes of main interest, a subsequent data-imputation technique (Bayesian imputation performed in the context of structural equation modeling) will be applied to obtain complete outcome data.

### Plans to give access to the full protocol, participant-level data, and statistical coding {31c}

The protocol has been registered at the ClinicalTrial.gov registry. The data will be shared only upon reasonable request to be evaluated by the PI.

## Oversight and monitoring

### Composition of the coordinating center and trial steering committee {5d}

The trial will be arranged and implemented under the responsibility of the IRCCS Istituto Centro San Giovanni di Dio Fatebenefratelli of Brescia. Three units of this Institute will carry out the study: the Unit of Psychiatry, the Unit of Biological Psychiatry, and the Service of Statistics. The coordinating center of the trial will be the Unit of Psychiatry. The PI will organize monthly meetings with the researchers’ staff to monitor the progress of the trial and to check if the plan is being followed. The PI will be responsible for preparation of the protocol, revisions, and periodic study reports. The PI will send annual reports about the study’s progress to the Ethics Committee. Moreover, two reports about project activity will be sent to the Ministry of Health at the midpoint and end of the project. The data management team will include the PI and the Service of Statistics. The data management team will assure quality data checks and maintenance of the database. Given the low risks added by the study, an independent oversight committee will not be established.

### Composition of the data monitoring committee, its role and reporting structure {21a}

Given the minimal risks of the present trial, a formal data monitoring committee will not be established. However, following local standards, data monitoring will be performed by an independent local ethics committee (Comitato Etico IRCCS San Giovanni di Dio, Fatebenefratelli, Via Pilastroni, 4 Brescia) that will oversee the study’s progress through periodic reports.

### Adverse event reporting and harms {22}

Given the type of research, specific plans for severe adverse events (SAEs) have not been formulated since neither SAEs are anticipated. Based on a literature review, no study reported harm for students who participated.

### Frequency and plans for auditing trial conduct {23}

The PI will organize monthly meetings with the researchers’ staff to monitor the progress of the trial and to check if the plan is being followed. The PI will send annual reports about the study’s progress to the Ethics Committee with the following information: the number of recruited participants, the number of participants who completed the study, the number of refusals, the number of drop-outs.

### Plans for communicating important protocol amendments to relevant parties (e.g., trial participants, ethics committees) {25}

The Ministry of Health and Ethics Committee will be notified of any changes to the protocol. After approval, a copy of authorized changes to the protocol will be uploaded to the trial registration site.

### Dissemination plans {31a}

The results will be presented at international scientific congresses and published in international scientific journals.

## Discussion

The present trial will contribute to further evidence on the use of an intervention with a strong theoretical foundation in a school setting in Italy. This is especially relevant since, to our knowledge, outside the USA, there is only one project from Ireland that applies DBT STEPS-A [60].

For the present trial, we will include in the intervention only some of the skills of the DBT STEPS-A program. Our choice was based on the consideration that the full 30-lesson program could be applicable only as an optional-choice course. Thus, to avoid self-selection bias and to be in line with a primary prevention approach, we preferred a shortened version of the program to be delivered to all students. Our purpose is also to gather evidence on the feasibility and outcomes of DBT-based interventions within the school environment, as well as to spread knowledge and awareness of mental health issues among teachers. In a recent study conducted in Ireland [60], the authors reduced the program’s length (from 30 to 22 lessons) to adapt it to the Irish school context; however, schools faced challenges in delivering the program, as only 2 out of the 8 participants completed the entire program (65). Although our intervention does not cover the entire DBT STEPS-A, it could be a valid strategy to develop a mental health-promoting culture in a school context, thus accomplishing the idea “to consider schools as part of a wider network involved in guaranteeing mental health in children and adolescents in local communities” [61].

The second aim of the study is to achieve a better understanding of ED. This is relevant since ED is a complex construct including emotional, cognitive, and behavioral components. The most common method to gauge ED is self-report questionnaires, mainly the DERS [4]. In the present study, we will include a comprehensive assessment that will evaluate both the use of emotional regulation skills and the frequency of dysfunctional behaviors as potential proxy variables of ED. Moreover, questionnaires focusing on depression, anxiety, family functioning, school climate, and stressful events will allow us to establish the influence of clinical and social factors as potential determinants of ED.

According to the biosocial model [20], ED implies the presence of biological vulnerability, which refers to a specific pattern of emotional processes characterized by high reactivity to external stimuli, high intensity of experienced emotions, and a slow return to baseline [20]. This pattern is directly associated with the expression of emotions (i.e., overt behaviors). Indeed, the complex phenomenon of emotions has neural substrates in bottom-up subcortical brain regions, while emotional regulation is mostly assured by top-down cortical brain networks [31, 41]. When high emotional reactivity is coupled with impairment in top-down control pathways, psychopathology is likely to occur [41]. Among the neurobiological processes underlying such regulation, stress may play a key role given its ability to directly influence cerebral circuits implicated in emotional processing [34]. Salivary cortisol is the most widely used method to assess HPA axis (dys)function. However, cortisol is only one of the products of HPA axis activation. In the present study, we will assess both cortisol and other inflammation mediators, such as cytokine levels. This will contribute to overcoming a limitation of the studies available, as they primarily focused on cortisol levels only. Moreover, it will be possible to better understand the mechanisms through which ED operates [40]. Given the paucity of studies on adolescents, data from the present trial could be particularly informative, as they will make it possible to accumulate knowledge about the relationship between HPA axis profiles in the general population of adolescents.

## Trial status

The protocol has been registered at ClinicalTrials.gov with the identifier number NCT04349709. Recruitment started in November 2019 and should be complete by November 2022.

### Authors’ contributions {31b}

LP is the principal investigator; she conceived of the study along with LRM, ML, RR, and AC. AC and NL helped to develop the protocol by providing their expertise about biological and molecular mechanisms. CF and AM will provide their statistical expertise in the clinical trial design. LP and SM will assure data collection and data entry, and coordinated and provided the intervention. VZ and AC will guarantee proper biological sample collections, measurements of biological variables, and bioinformatic analyses. All authors have helped to refine study protocol and approved of the final manuscript.

## Abbreviations

ED: Emotional dysregulation
SEL: Socioemotional learning
HPA: Hypothalamic-pituitary-adrenal axis
DBT: Dialectical Behavioral Therapy
DBT STEPS-A: Skills Training for Emotional Problem Solving for Adolescents
